# Achieving self-awareness through film screening “Twin Peaks” By D. Lynch as an example of mindfulness meditation

**DOI:** 10.1192/j.eurpsy.2024.1373

**Published:** 2024-08-27

**Authors:** J. K. Nowocień, N. Szejko

**Affiliations:** Department of Medical Ethics and Palliative Medicine, Medical University of Warsaw, Warsaw, Poland

## Abstract

**Introduction:**

Transcendental cinema, distinguished from slow cinema by Paul Schreader, draws on the philosophy of existentialism and depicts the complexity of the human psyche using psychoanalytic tools. We claim that through the use of special procedures, the projection of transcendental cinema essentially becomes a meditation session in the spirit of mindfulness, which has been proven to alleviate and cure more than just neuropsychiatric ailments.

**Objectives:**

The purpose of this work is to demonstrate the similarity between mindfulness philosophy and transcendental cinema. We believe that the assumptions of both currents are so similar that we can treat the film screening in the category of a meditation session. Thus, we arrive at a situation in which we not only watch the protagonist developing his own consciousness in accordance with the mindfulness philosophy (also following the path of psychoanalysis), but also we, as viewers, develop self-awareness.

**Methods:**

We analyze D. Lynch’s Twin Peaks series in accordance with Paul Schrader’s understanding of ‘transcendental cinema’. In addition, we use the scientific achievements of classical psychoanalysts, analyzing the metaphysical world of the characters in accordance with this trend. Using J. Kabat Zinn’s scientific publications, we analyze cinema in terms of a meditation session.

**Results:**

Participation is crucial; in meditation and in the transcendental cinema. Mindfulness means focusing on the emotions and feelings experienced at a given moment, on what comes to us, what we experience. Transcendental cinema using specific formal and narrative tools (e.g. extended scenes, no cuts, etc.) forces us to actively participate. Transcendental cinema fulfills the tenets of mindfulness, and during the screening we undergo a meditation session. What’s more, this style in cinema allows an in-depth exploration of the psyche, it brings us closer to the metaphysical, emotional dimension of humanity what develops in us the ability to understand the psyche of others, as well as our own.

**Conclusions:**

We claim that the similarity between the philosophy of mindfulness and transcendental cinema allows us to treat a film screening as a meditation session. Cinema enriches us not only with knowledge about disorders and the therapeutic process, but is in itself a supportive tool - screening can allow viewers to deepen their awareness and improve their health. What is more, David Lynch’s work brings us closer to exploring the human psyche and the individualization of inner experiences, while also showing us what influence transcendental meditation has on characters and what happens when they undergo a kind of therapy; in the spirit of psychoanalysis or mindfulness philosophy.

**Disclosure of Interest:**

None Declared

